# Surface and Grain Boundary Coating for Stabilizing LiNi_0.8_Mn_0.1_Co_0.1_O_2_ Based Electrodes

**DOI:** 10.1002/cssc.202400272

**Published:** 2024-08-12

**Authors:** Zahra Ahaliabadeh, Ville Miikkulainen, Miia Mäntymäki, Seyedabolfazl Mousavihashemi, Lide Yao, Hua Jiang, Simo Huotari, Timo Kankaanpää, Tanja Kallio, Mattia Colalongo

**Affiliations:** ^1^ Department of Chemistry and Materials Science (CMAT) School of Chemical Engineering Aalto University Espoo 02150 Finland; ^2^ Department of Chemistry University of Helsinki Helsinki 00014 Finland; ^3^ Department of Applied Physics School of Science Aalto University Espoo 02150 Finland; ^4^ Department of Physics University of Helsinki Helsinki 00014 Finland; ^5^ Umicore Finland Oy Kokkola 67101 Finland; ^6^ European Synchrotron Radiation Facility 71 Avenue des Martyrs 38000 Grenoble France

**Keywords:** atomic layer deposition, cracking, grain boundary, lithium titanate coating, nickel-rich positive electrode, phase transitions

## Abstract

The widespread use of high‐capacity Ni‐rich layered oxides such as LiNi_0.8_Mn_0.1_Co_0.1_O_2_ (NMC811), in lithium‐ion batteries is hindered due to practical capacity loss and reduced working voltage during operation. Aging leads to defective NMC811 particles, affecting electrochemical performance. Surface modification offers a promising approach to improve cycle life. Here, we introduce an amorphous lithium titanate (LTO) coating via atomic layer deposition (ALD), not only covering NMC811 surfaces but also penetrating cavities and grain boundaries. As NMC811 electrodes suffer from low structural stability during charge and discharge, We combined electrochemistry, operando X‐ray diffraction (XRD), and dilatometry to understand structural changes and the coating protective effects. XRD reveals significant structural evolution during delithiation for uncoated NMC811. The highly reversible phase change in coated NMC811 highlights enhanced bulk structure stability. The LTO coating retards NMC811 degradation, boosting capacity retention from 86 % to 93 % after 140 cycles. This study underscores the importance of grain boundary engineering for Ni‐rich layered oxide electrode stability and the interplay of chemical and mechanical factors in battery aging.

## Graphical abstract

Lithium‐ion battery high‐capacity Ni‐rich layered oxides electrode materials, like LiNi_x_Mn_y_Co_z_O_2_, suffer from capacity loss and reduced working voltage during battery cycling. To overcome, lithium titanate coating penetrating to grain boundaries is applied through atomic layer deposition to enhance structural and chemical stability and thus reducing degradation and improving capacity retention

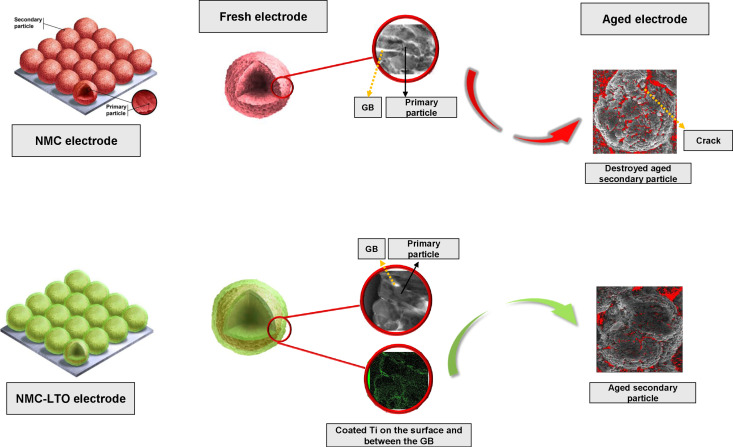

## Introduction

The large‐scale deployment of electric vehicles (EVs) and stationary battery systems creats urgent requests for rechargeable batteries with higher energy/power density and longer lifetime.[^1^, ^2^, ^3^, ^4^, ^5^] Li‐ion battery (LIB) demand is growing tremendously as they simultaneously satisfy the requirement of high energy and power density. Due to the high capacity, high energy density, and low cost, Ni‐rich cathode materials, especially NMC family materials (LiNi_x_Mn_y_Co_z_O_2_ (NMC), x>0.5, 0<x,y,z<1, x+y+z=1), are very promising positive electrode materials meeting the demanding requirement for EVs.[^1^, ^6^] The most effective approach to further increase the energy density and reduce production costs of LIBs is to increase the Ni content while lowering the Co content in NMC materials. However, the practical implementation of positive electrode materials with high Ni content is hampered by severe capacity fading and structural stability issues induced by long‐term cycling. Increasing Ni content in NMC can lead to an increase in cation mixing due to the similar size of *Ni* ^2+^ (0.69 Å) and *Li* ^+^ (0.76 Å). Migration of *Ni* ^2+^ into the Li vacancies further induces surface reconstruction from the layered to inactive disordered spinel or rock‐salt structure hindering lithium‐ion diffusion.[^7^, ^8^, ^9^] During cycling, the Ni‐rich layered NMC materials undergo large anisotropic volume change, compromising the mechanical stability and generating micro cracks. These cracks propagate along particle grain boundaries (GBs), causing the fragmentation of the NMC secondary particles and consequently leading to rapid capacity fading.^[10]^ GBs refer to the spaces between the needle shaped primary particles which aggregate to form the spherical secondary particles. The infiltration of liquid electrolyte into these GBs reduces grain‐to‐grain contacts and during the electrode volume changes generates intergranular craks. This significantly increases the interphase area between the positive electrode active material and the electrolyte, which promotes electrolyte decomposition at high potential.[^8^, ^11^]

All these unfavorable factors cause deterioration of the Ni‐rich material and compensate for the advantage of higher energy density when cycled to high voltages. Batteries based on nickel‐rich ${\mathrm{LiNi}}_{0.8}{\mathrm{Co}}_{0.1}{\mathrm{Mn}}_{0.1}O_{2}$ (NMC811) materials do not meet service life expectations due to continuous capacity loss and reduced working voltage. This is a significant challenge that must be resolved for the development of better Lithium‐ion batteries. Improved stability and performance of NMC811‐based batteries are being explored to overcome this challenge.^[12]^

Heenan et al. showed that cracking may appear already during electrode fabrication. They reported that once the active material is printed into a sheet and calendared, the fabrication‐induced cracking can be severe, with approximately one‐third of particles being defective before operation (Figure S1). These defects can grow before cycling or even assembly of the cells, which leads to the degradation of the electrode material at the electrode‐electrolyte interface, by the electrolyte diffusing into the cracks and dissolving the transition metals (TM).^[13]^ Applying a surface coating with high ionic conductivity is considered an effective strategy to stabilize the electrode/electrolyte interface. The surface coating can prevent direct contact between cathode active materials and a liquid electrolyte, mitigate parasitic reactions, TM dissolution, and even accommodate lattice expansion/contraction during the charge/discharge processes.[^14^, ^15^] Wet chemistry has been widely used to apply coatings on NMC particles but does not allow controlling of the coating quality and removing undesired residuals completely.^[16]^ In the past decade, atomic layer deposition (ALD) has emerged as an alternative method, which uniquely enables the synthesis of high‐quality conformal and uniform films over either electrode particles or electrode films.[^17^, ^18^, ^19^] In addition, ALD can be used at low process temperatures (less than 200 °C) in comparison to solid‐state methods and allow accurate control over film growth at the atomic level.^[19]^ To date, many different coatings have been reported for modifying NMCs via wet chemistry and ALD, including metal oxides such as ${\mathrm{Al}}_{2}O_{3}$ ,^[20]^ ZrO_2_,^[21]^ TiO_2_,^[22]^ WO_3_,^[23]^ and lithium metal oxides,^[19]^ phosphates,^[11]^ and fluorides.^[24]^ Li‐containing metal oxides have great potential as an artificial cathode electrolyte interface (CEI) owing to high conductivity, and high interface energy with Li.[^16^, ^19^, ^24^] Lithium titanate (LTO), due to its low dimensional change with altering lithium content, has been rarely investigated as a *Li* ^+^‐conductive coating for NMC811 positive electrode materials.[^19^, ^25^, ^26^, ^27^, ^28^, ^29^, ^30^, ^31^, ^32^, ^33^, ^34^, ^35^, ^36^, ^37^]

Recently, Li et al.^[38]^ demonstrated LTO surface coating through the wet chemistry method on NMC811 particles. The LTO coating can effectively protects the surface of NMC811 particles from the corrosive side reactions and provides better *Li* ^+^ transport channel accelerating the lithium diffusion.^[38]^ In our previous study,^[19]^ we examined the benefits of LTO coatings compared to TiO_2_ coatings on NMC622. The findings demonstrated that the rate capability of NMC622 coated with LTO surpassed that of NMC622 coated with TiO_2_ and uncoated NMC622. This improvement is attributed to the superior lithium diffusivity and electronic conductivity of LTO over TiO_2_. Besides, the results showed that the electrochemical behavior of Ni‐rich NMC622 is enhanced by the LTO coating at relatively high potentials (X≥4.3 V). It should be noted that both surface aging and mechanical failure occur simultaneously during battery operation^[12]^ and thus the benefit of the LTO coating on the NMC622 electrodes is mitigating electrolyte oxidation and reducing electrode corrosion. In addition, the LTO coating protects the NMC622 electrode in multiple aspects by improving the mechanical integrity of the NMC622 particles, stabilizing the interface between the NMC622 particles and electrolyte, and mitigating the irreversible structural phase transition.

Recently, Cheng et al.^[11]^ proposed ${\mathrm{Li}}_{3}{\mathrm{PO}}_{4}$ coating to tailor the GBs of NMC secondary particles. The results showed that the coating prevents the penetration of liquid electrolytes into GBs and increases structural stability. Also, ionic conductivity incremented and, thus intergranular crack formation is successfully suppressed. Therefore, superior cyclability and less capacity loss should be achieved with GB coatings during the long‐term cycling.[^39^, ^40^]

Inspired by our previous work with NMC622,^[19]^ in this study we investigate LTO coating for tackling the issues of NMC811 featured by distinctly different electrochemical behavior compared to NMC622.^[41]^ We have coated Ni‐rich NMC811 prefabricated electrodes with LTO using ALD to demonstrate the effect of surface coating and GB engineering on the electrochemical performance of these electrodes. It is found that suppressing intergranular crack formation is crucial for achieving superior cycling stability. However, recent research on combining surface protection and mechanical enhancement for prefabricated electrodes is rare and the protection mechanism not elaborated. By utilizing advanced characterization tools, such as scanning transmission electron microscope (TEM), opernado dilatometry, and opernado X‐ray diffraction, we gain deep insight into the structure‐property relationship in terms of degradation and material disintegration. The achieved results reveal that the structural and chemical stability of NMC811 is enhanced by the LTO coating which produces benefits in protecting NMC811 during cycling. Consequently, this work is significant, as it addresses the mechanochemical protection offered by the LTO coating for enhancing NMC811 prefabricated electrodes performance which has not been well investigated previously and opens a corner for evaluating coating effects in general.

## Results and Discussion

### Structural Characterization

**Figure 1:**
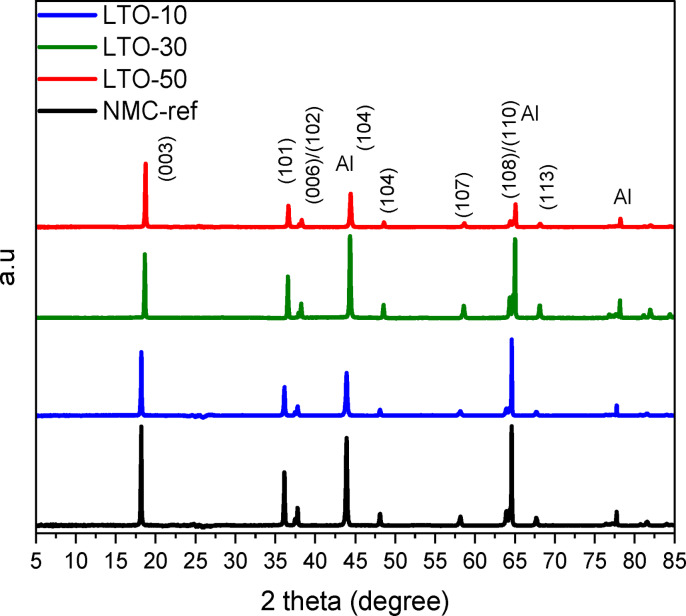
XRD diffraction patterns (from down to top) of the NMC‐ref, NMC‐LTO‐10, NMC‐LTO‐30 and NMC‐LTO‐50 samples.

Based on our previous experience, LTO coating synthesized via ALD^[19]^ for Ni‐rich NMC622 enhances electrochemical behavior even at relatively high potentials (X≥4.3 V). Inspired by that work, we investigate in this study LTO coating for tackling issues related to NMC811 which is featured by distinctly different electrochemical behavior compared to NMC622. LTO was deposited directly on the surface of the NMC811 electrodes (for details see Experimental section) using the ASM F120 ALD system following a previously reported method.^[19]^ The uncoated NMC and LTO‐coated samples were characterized by powder X‐ray diffraction. No appreciable differences in terms of crystallinity and phase purity are observed, as shown in Figure 1, suggesting that the LTO coating does not alter the NMC811 bulk material structure.^[42]^ All the typical XRD peaks of NMC811 are detected for the uncoated and coated NMC811 electrodes, while distinct splitting of the (006)/(102) and (108)/(110) peaks demonstrate a well‐developed layered hexagonal *α*‐NaFeO_2_ structure. No diffraction peaks related to the LTO structure are detected as expected based on the nano‐scale thickness and presumed amorphous nature of the ALD‐coated film. This is consistent with our previous study,^[19]^ in which LTO films were deposited by ALD and remained amorphous in the deposition temperature of 200 °C.

**Figure 2:**
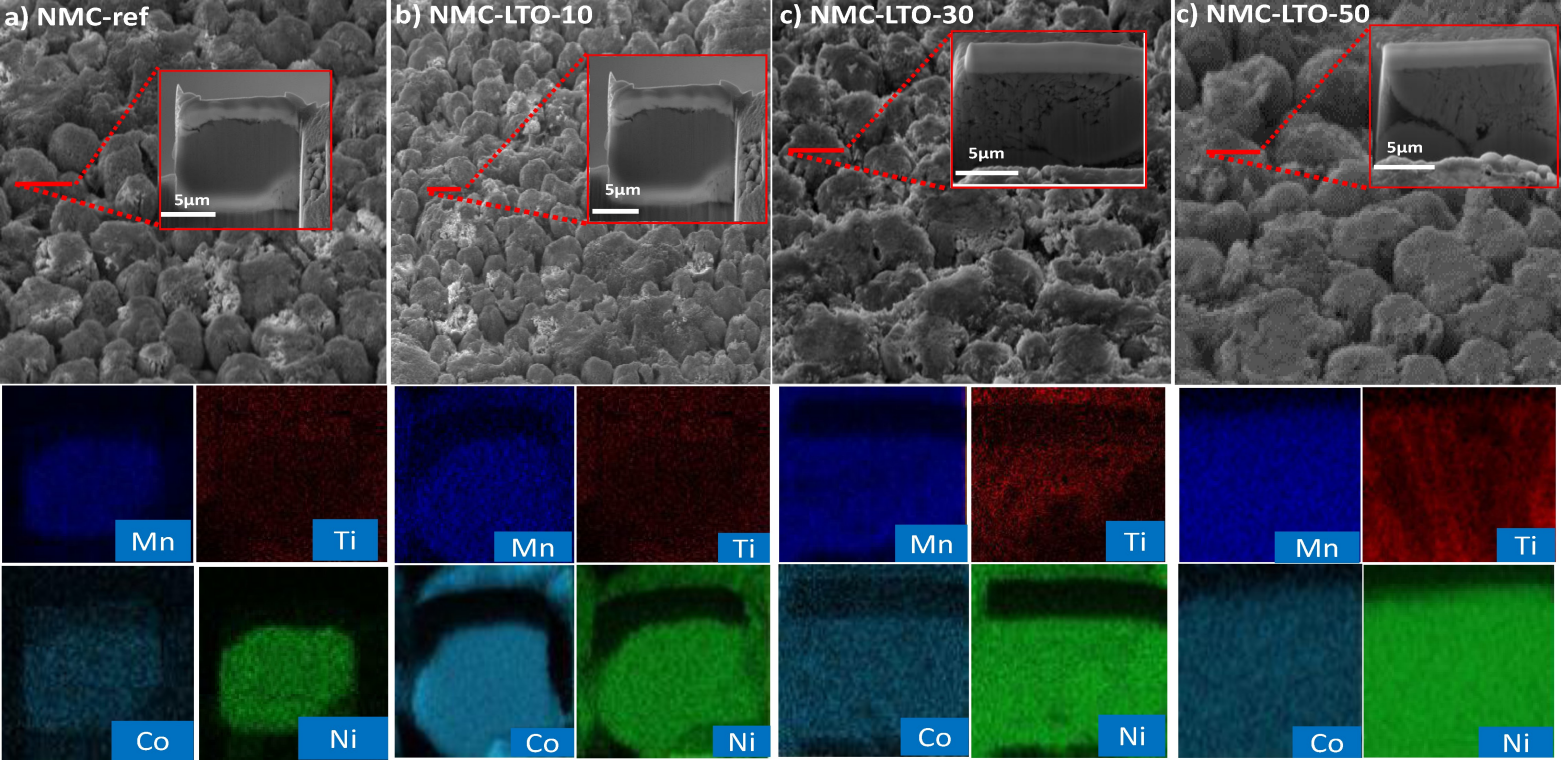
A SEM image, FIB‐SEM image, and corresponding EDS mapping at the selected area for the a) NMC‐ref, b) NMC‐LTO‐10, c) NMC‐LTO‐30, and d) NMC‐LTO‐50 sample.

As depicted in Figure 2, the NMC811 electrodes are coated with LTO and the coating is also infused into the NMC811 particles and cavities. When comparing the morphologies of the NMC‐ref and NMC‐LTO‐10 electrodes illustrated in Figure 2a and 2b, respectively, the morphology of the NMC‐LTO‐10 does not exhibit any evident changes. Figure 2c and d also reveal a similar morphology for NMC‐LTO‐30 and NMC‐LTO‐50 to the NMC‐ref sample owing to the thin nature of the ALD coatings.[^43^, ^44^] However, Figure 2 SEM‐FIB images with elemental mappings of the cross‐section samples visualize the difference between the samples clearly. The elemental mapping of the NMC‐ref and LTO‐coated NMC electrodes in Figure 2a–d confirms that Ni, Mn, and Co are distributed uniformly in all samples whereas Ti covers the surface and cavities of NMC‐LTO‐30 and NMC‐LTO‐50. The enlarged views of the Ti mapping are shown in yellow boxes, in order to show the diffusion of Ti into the electrode GBs during the ALD process. The elemental mapping of NMC‐LTO‐10 does not show a visible distribution of Ti because for this low coating thickness, the Ti amount remains below the SEM‐EDS detection limit. However, Ti detection on the surface of NMC‐LTO‐10 is shown by the *TEM study* in the following paragraph. In particular, these SEM‐EDS observations confirm that for the NMC‐LTO‐30 and NMC‐LTO‐50 samples, Ti has successfully diffused into the porosity of the secondary particles and enriched at GBs (Figure 2c–d). The coating of cracks formed during the electrode fabrication, due to issues with mechanical integrity, is highly important. Those cracks can lead to proceeding fragmentation of active materials, which not only causes poor electronic conduction but also expose fresh surfaces to the electrolyte. Crack formation is also considered as one of the main causes of cell performance decay, and hence the protective coating of these surfaces can greatly enhance the electrolyte cycle life.[^14^, ^45^, ^46^, ^47^]

**Figure 3:**
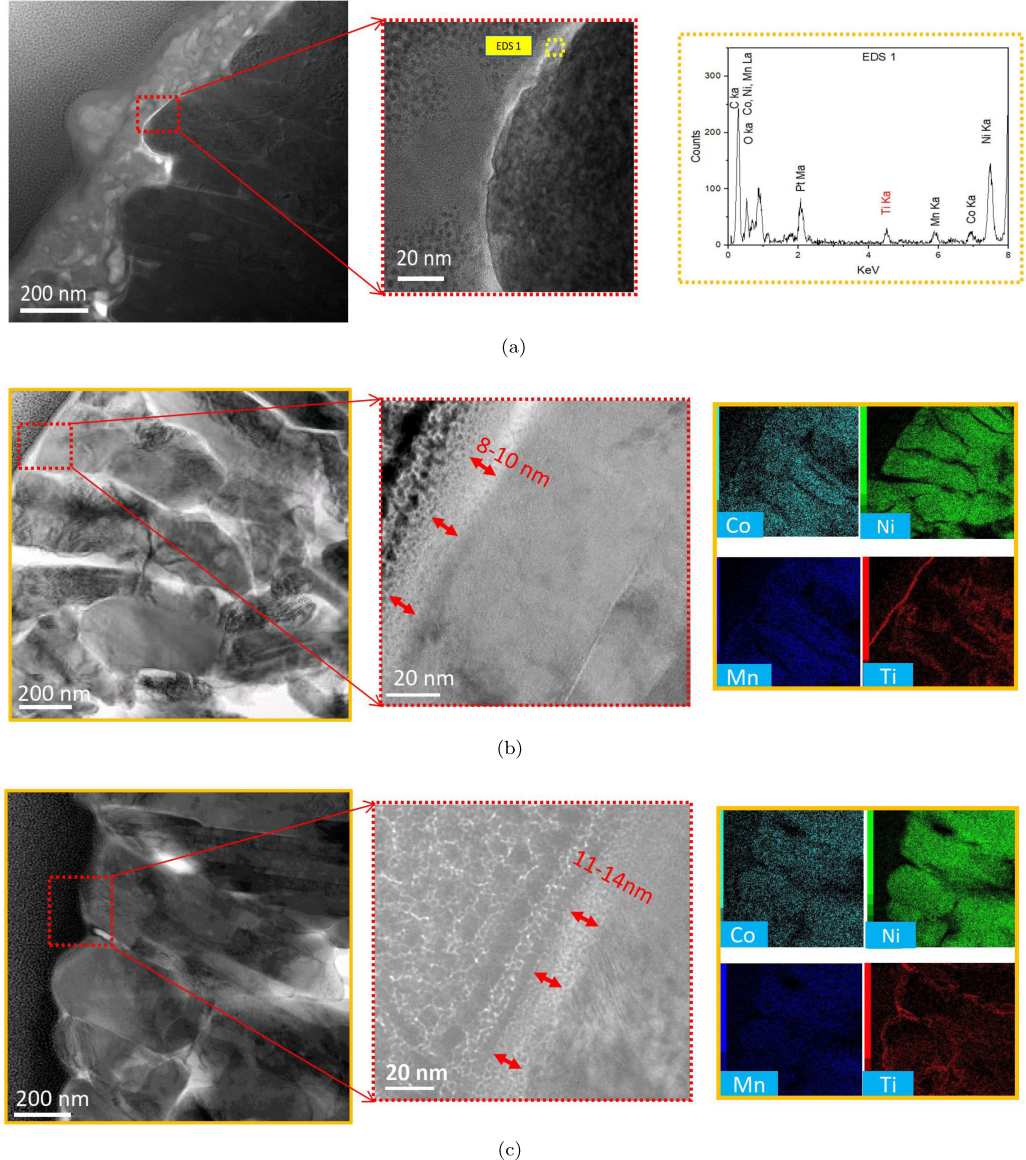
Dark field STEM images with EDS analysis and embedded thickness. Representative images for the a) NMC‐LTO‐10, b) NMCLTO‐30, and c) NMC‐LTO‐50 sample.

A cross‐sectional high‐resolution TEM study has been performed to further reveal details of the coatings. The sample preparation for the STEM elemental mappings to observe the selected area on the electrode surfaces has been explained in detail in our previous work.^[19]^ For the samples with the thicker coatings, the TEM coupled with EDS evidence a uniform Ti layer covering the surface of the NMC811 particles (Figure 3). NMC‐LTO‐30 and NMC‐LTO‐50 exhibit an amorphous LTO coating formed on the surface of the primary NMC811 particles which is consistent with the above SEM results and further verifies that Ti is incorporated into the GBs. The surfaces of NMC‐LTO‐30 (Figure 3b) and NMC‐LTO‐50 (Figure 3c) are covered by a thin layer, indicating a coating with different thicknesses of 8–10 nm and 11–14 nm, respectively. An evident LTO layer cannot be observed on NMC‐LTO‐10 (Figure 3a) though the EDS analysis confirms the presence of Ti on the surface (Figure S2). This is attributed to the very low thickness of LTO (x≤2 nm) and accordingly, the Ti‐based coating is plausibly located within the NMC surface porosity. The uniformity of the LTO coating has been confirmed by TEM‐EDS measurements on different locations at the NMC‐LTO‐10 surface which shows a similar Ti coverage across the NMC811 particle (Figure S2).

Different LTO coating thicknesses, whether on the surface or in the cavities, enable analyzing the coating effects on the electrochemical performance of the NMC811 electrode. The surface coating and GBs engineering have been earlier found to effectively enhance cycling stability due to suppressed intergranular cracks.[^14^, ^46^] Enhancing the surface properties of the NMC811 secondary particles and GBs can increase the cell cyclability and capacity, especially for cells cycled at high cut‐off voltages.[^12^, ^19^]

### Electrochemical Characterization

**Figure 4:**
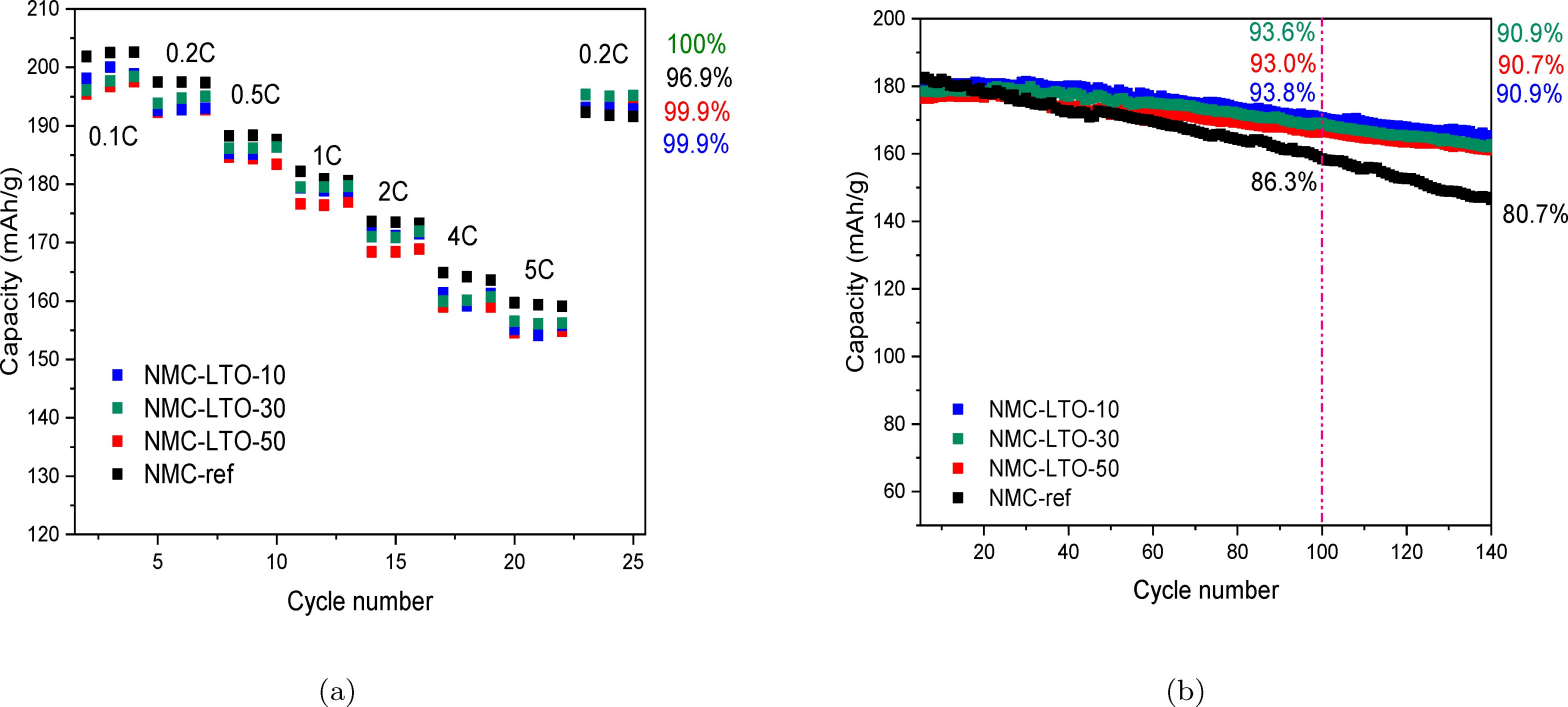
Electrochemical performance of the NMC‐ref, NMC‐LTO‐10, NMC‐LTO‐30 and NMC‐LTO‐50 electrodes in half cells. (a) Rate capability of the NMC‐ref and LTO‐coated NMC811 electrodes at various current densities in the voltage range of 3.0–4.4 V. (b) Long‐term cyclability (charged and discharged at 1 C) of the NMC‐ref, NMC‐LTO‐10, NMC‐LTO‐30 and NMC‐LTO‐50 electrodes in the voltage range of 3.0–4.4 V.

We conducted electrochemical studies for the uncoated and three samples with different LTO coating thicknesses to compare their effect on rate capability and cycling performance. For all samples, the coating improves the cycling stability suggesting that this surface and GB engineering enables efficient protection. Figure 4a shows the rate capability of the NMC‐ref and LTO‐coated NMC811 electrodes in half cells which are charged/discharged at different C‐rates (0.1, 0.2, 0.5, 1, 2, 4, and 5C) for 3 cycles at 3.0–4.4 V. The rate capabilities of the NMC‐ref and the coated NMC811 electrodes are comparable at different C‐rates, implying that the LTO coating does not block the *Li* ^+^ diffusion.[^19^, ^48^] The rate capability results indicate that the positive effects of the LTO coatings became evident with the last cycling step at 0.2C: Higher capacity retention leads to improved capacities for the coated NMC811 electrodes in comparison to the uncoated ones.

The long‐term cycling results, 100 cycles at 1C (1C=200 mA/g) in the range of 3.0–4.4 V in half cells, indicate that the applied surface and GB engineering enables nearly equal improvement (Figure 4b) with the capacity retention of 93.0 %, 93.6 %, 93.8 %, and 86.3 % for NMC‐LTO‐10, NMC‐LTO‐30, NMC‐LTO‐50, and NMC‐ref, respectively. Cycling the samples for longer, up to 140 cycles, demonstrates mitigated capacity decay and voltage fading for the coated samples (2 % decrease) in comparison to the NMC‐ref (6 % decrease) as presented in Figure 4b. Along with the faster capacity fading, the NMC‐ref electrode is detected to experience a larger discharge voltage drop than the NMC‐LTO‐10, NMC‐LTO‐30, and NMC‐LTO‐50 electrodes. Fig. 3S illustrates the evolution of the average discharge voltage calculated by dividing the discharge energy by the discharge capacity.^[18]^ The corresponding values equal to 0.32, 0.15, 0.14, and 0.16 V for NMC‐ref, NMC‐LTO‐10, NMC‐LTO‐30, and NMC‐LTO‐50 after 140 cycles, respectively.

**Figure 5:**
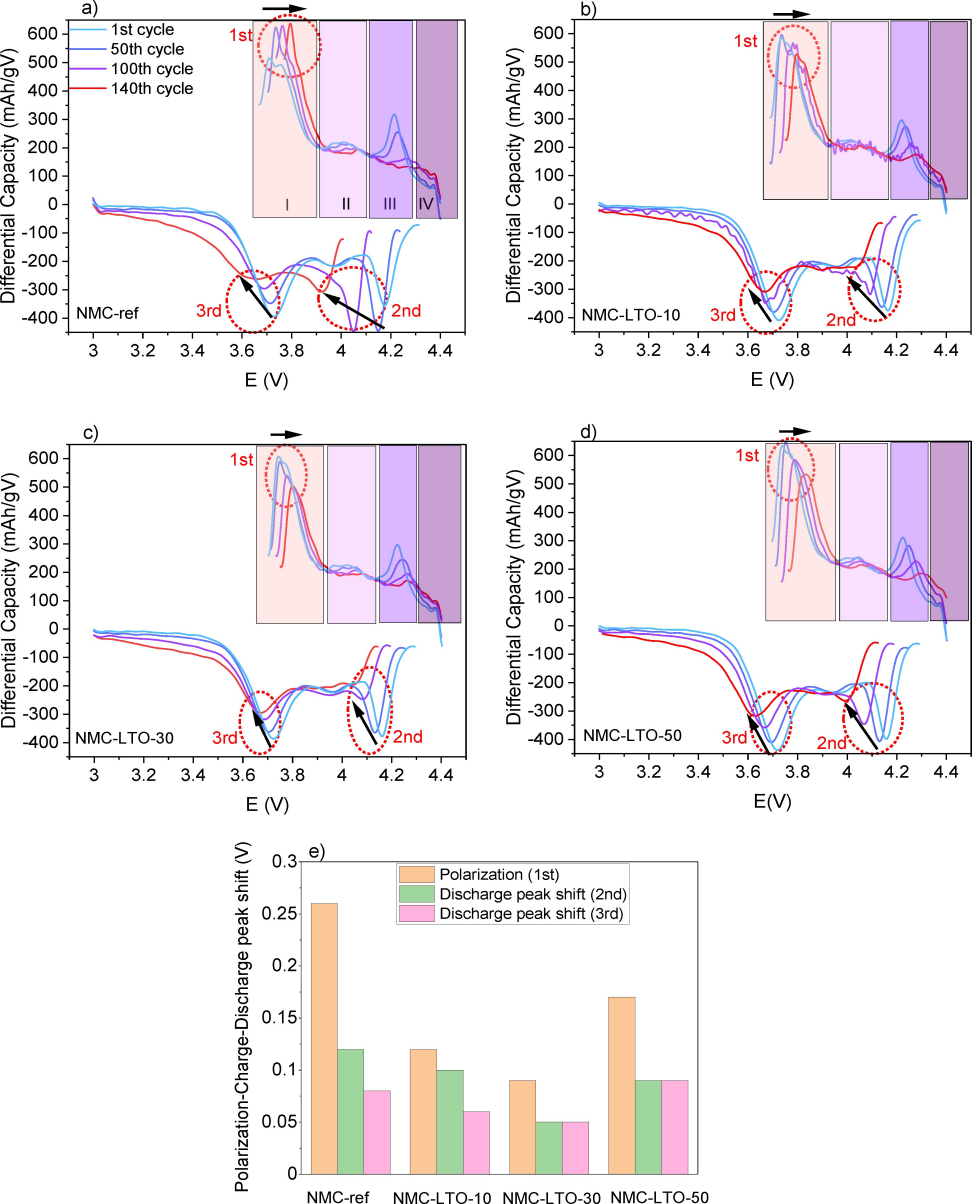
Differential capacity plots of charge and discharge curves of the long‐term cycling sequence of (a) NMC‐ref, (b) NMC‐LTO‐10, (c) NMC‐LTO‐30, (d) NMC‐LTO‐50. (e) Polarization evolution of the 1st (charge), 2nd, and 3rd (discharge) between the first and the last cycle (ΔP) of the long‐term cycling sequence for the samples.

To get deeper insight, we further analyzed the differential capacity versus voltage (dQ/dV) , as illustrated in Figure 5 for the NMC‐ref, NMC‐LTO‐10, NMC‐LTO‐30, and NMC‐LTO‐50 samples. Four regions are discerned, labeled as regions I, II, III, and IV in Figure 5. Regions I, II, and III contribute the majority of the capacity, while very limited capacity is achieved in the last region without any redox peaks. These peaks are also observed for ${\mathrm{LiNiO}}_{2}$ chemistry, where they are coupled with distinct first‐order phase transitions meaning that (de)intercalation leads to a two‐phase coexistence which differs in composition, lattice parameters, and their symmetry.^[50]^ To verify whether these phase transitions also occur in the NMC‐ref and LTO‐coated NMC811 materials, operando XRD experiments were performed to reveal their structural changes during cycling (see chapter 3.4 for discussion). For all the samples, the position of the three peaks in the regions I, II, and III during charge are found to shift from 3.70 to 3.80, 4.0 to 4.1, and 4.2 to 4.3 V vs. $Li^{+}/Li$ , respectively, from 1st to 140th cycle which is consistent with previous reports.[^50^, ^51^] In general, a dQ/dV plot reveals the extent of polarization which is closely linked to interfacial properties. Thus, the shifts in the peak positions indicate an increase in the cell polarization during cell cycling which is caused by interfacial side reactions.^[27]^ The polarization behavior of the individual samples in the three different areas of the dQ/dV curves is demonstrated and compared in Figure 5e. The oxidation and reduction peaks measured for the NMC‐ref electrode show clearly much larger shifts than those for the LTO‐coated‐NMC811 electrodes during 140 cycles. Based on these results, we can conclude that the NMC‐ref electrode suffers from a larger polarization increase during charge/discharge cycling when compared to the coated ones.[^19^, ^52^] The higher polarization can be caused by the crack formation, thick or deteriorated CEI, and/or irreversible phase transitions.[^27^, ^52^] When the measurement continues up to 140 cycles, the intensity of the oxidation peak at 4.2 V decreases considerably more for the NMC‐ref sample compared to the LTO‐coated‐NMC811 samples. This decrease is likely due to the structural degradation that occurs during the lithiation‐delithiation process in the NMC‐ref electrode, while the LTO coating improves the stability of the NMC811 electrodes.

**Figure 6:**
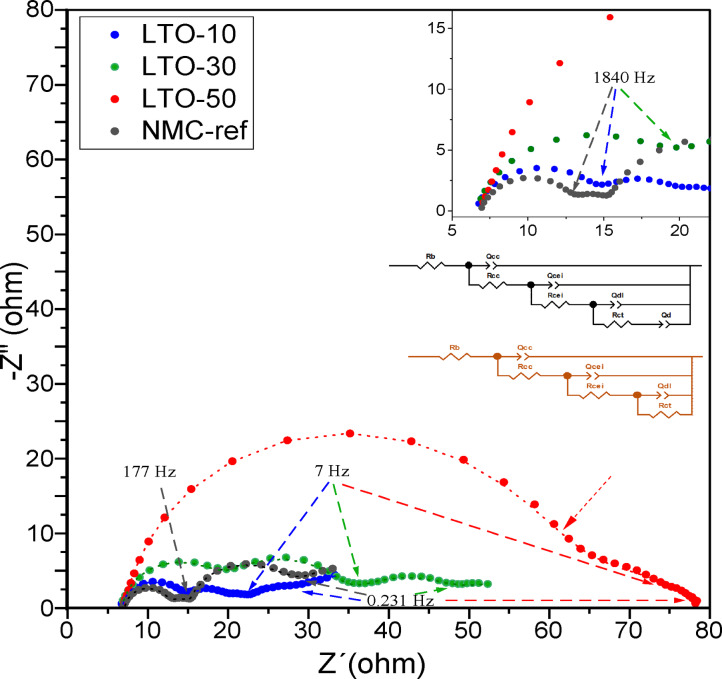
EIS measurements, Nyquist plots, and fitting data for the NMC‐ref, NMC‐LTO‐10, NMC‐LTO‐30, and NMC‐LTO‐50 electrodes with an amplitude of 5.0 mV over a frequency range of 100 kHz to 0.01 Hz after the formation cycles. Each cell was charged to SOC 50 % before the EIS measurements. The corresponding equivalent circuits have been plotted as an embedded figure in the Nyquist plot.

Electrochemical impedance spectroscopy (EIS) was performed to investigate the effect of the coating thicknesses on the electrochemical kinetics of the LTO‐coated electrodes. Nyquist plots of the NMC‐ref and LTO‐coated NMC811 electrodes are presented in Figure 6. Three semicircles can be discerned for all samples and a tail at the lowest frequencies only for the NMC‐ref, NMC‐LTO‐10, and NMC‐LTO‐30 samples. The corresponding equivalent circuits are plotted as an embedded figure in the Nyquist plot. The lower one represents the EIS model without the diffusion tail for NMC‐LTO‐50 while the upper one is relevant for the other samples. *Rb* corresponds to the ohmic resistance including the electrolyte, aluminum foil, electrode wires, and connectors. The first semicircle at high frequencies, demonstrated by *Rcc* and *Ccc* in the equivalent circuit model, is correlated to the transport of electrons from the current collector to the active material and the transport of electrons within the active material particle. The second semicircle in the high‐to‐medium‐frequency range is ascribed to the CEI which is evolving gradually during the formation cycles and includes the LTO as an artificial CEI. *Rcei* value shows the impedance of *Li* ^+^ migration through the CEI. The third semicircle in the medium‐to‐low frequency range represents the impedance of the charge transfer process at the interface of the electrode material and the electrolyte which is shown by *Qdl* and *Rct* elements in the equivalent circuit. The tail in the low‐frequency range is ascribed to the solid‐state diffusion of *Li* ^+^ in the active material structure.[^53^, ^54^, ^55^] The fitting results of the EIS data for the NMC‐ref and LTO coated NMC811 samples are shown in Table 1. Those indicate that the total resistance (*Rb*+*Rcc*+*Rcei*+*Rct*) of the samples after 10 formation cycles varies increasing with the LTO thickness. The highest total resistance of 79.3 Ohm is found for the NMC‐LTO‐50 sample compared to 45.5 Ohm for NMC‐LTO‐30 and 32.0 Ohm for NMC‐LTO‐10, and 27.0 Ohm for the uncoated NMC‐ref. These results are in agreement with the obtained overvoltage values for the samples during the galvanostatic charge‐discharge studies. The EIS fitting results for all the studied samples indicate that the *Rb* values are in the same range with a slight random variation, which relates to the experimental setup preparation. *Rcc* and *Rcei* values show an increment in the impedance with an increase in the LTO coating thickness. Thus, the NMC‐ref sample has the lowest *Rcc* (5.6 Ohm) and *Rcei* (3.0 Ohm) values among the samples. The electron transport from the current collector to the active material and electron transfer through CEI is impeded by LTO functioning as an extra insulator layer. Yet, the obtained charge transfer resistance values reveal that the lithium (de)intercalation performance is not significantly affected by the LTO coating as depicted in the rate capability measurements (Figure 4). As such, the results show that both NMC‐LTO‐10 and NMC‐LTO‐30 have comparable behavior and they do not hinder lithium or electron transfer through the CEI layer contrary to the NMC‐LTO‐50 sample.

**Table 1:** Electrochemical resistance of the NMC‐ref, NMC‐LTO‐10, NMC‐LTO‐30 and NMC‐LTO‐50 samples at SOC 50 %.

	NMC	LTO‐10	LTO‐30	LTO‐50
	Rb(Ohm)	Rcc(Ohm)	Rcei(Ohm)	Rct(Ohm)
NMC	7.2	5.6	3.0	11.2
LTO‐10	7.0	6.5	8.3	10.2
LTO‐30	6.9	13.0	15.6	9.9
LTO‐50	7.1	41.8	16.9	13.5

**Figure 7:**
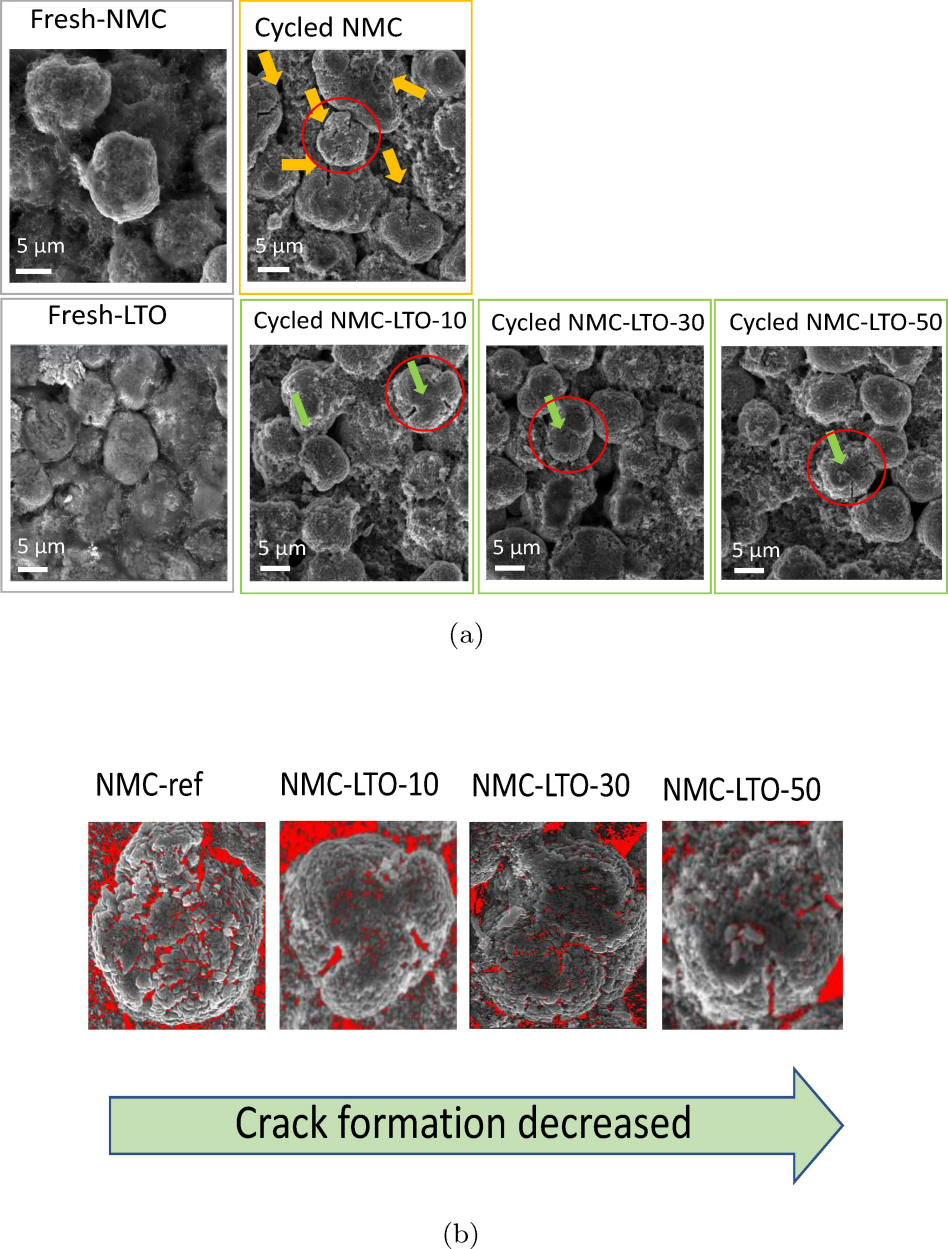
SEM images of the (a) NMC‐ref and NMC‐LTO‐30 and the postmortem SEM for the aged NMC‐ref, NMC‐LTO‐10, NMC‐LTO‐30 and NMC‐LTO‐50 after 300 cycles at 1 C in the voltage range 3.0–4.4 V in half cells. (b) Enlarged view of the selected secondary particles in (a) the NMC‐ref and coated NMC811 electrode after the cycling. The red area shows the cavities.

To further characterize the morphological and structural evolution of the NMC‐ref and LTO‐coated NMC811 electrodes after 300 charge‐discharge cycles, we employed a postmortem analysis of the aged sample by SEM. The 300‐cycle galvanostatic charge‐discharge results for the studied electrodes are shown in Figure S4. Figure 7 shows the NMC‐ref and NMC‐LTO‐30 fresh electrodes as well as NMC‐ref and NMC‐LTO‐10, NMC‐LTO‐30, and NMC‐LTO‐50 postmortem SEM results. We only show the NMC‐LTO‐30 fresh electrodes as no difference is observed with the used magnification between the morphology of the fresh coated samples because the LTO coating thickness is in the nm range. Based on the postmortem results, the cycled NMC‐ref particles clearly have numerous cracks produced on the particle surface (Figure 7a). However, the coated samples show higher stability during the cycling. Figure 7b shows the selected particles in Figure 7a for the NMC‐ref and LTO‐coated NMC811 electrodes to determine the evolution of porosity and cracks into the secondary particles during the cycling. The imaging software Image J was used to determine the morphology evolution of the secondary particles in the postmortem SEM images. It is clearly demonstrated that the crack generation intensity is reduced with the increasing coating thickness which indicates the effect of LTO as a protective layer for the NMC electrodes.[^12^, ^19^] Besides, Figure 7b demonstrates that NMC‐LTO‐50 and NMC‐LTO‐30 have a lower amount of cracks formed on their surface (due to the less stress and strain as shown in the following sections) which is attributed to the higher mechanical protection offered by the LTO coating.^[17]^ As confirmed in the long‐term cycling results, crack formation exposes intact primary particles to the electrolyte and thereby the local particle surface covered by the LTO layer can hinder parasitic reactions during the long‐term cycling.^[12]^ While NMC‐LTO‐50 is proven to induce higher overvoltage and CEI layer resistance, NMC‐LTO‐30 provides the same protection but with lower overvoltage and cell impedance. This work suggests that the cracks generated on the NMC811 particle surface and subsequently propagating into the structure are a major deleterious degradation mechanism in layered positive electrode structures.[^11^, ^12^] As such, based on the SEM postmortem analysis and electrochemical investigation, it can be concluded that NMC‐LTO‐30 can protect the surface of NMC811 while not blocking the Li path with thick CEI layer formation.

### Operando Dilatometry Analysis

Volume variations are related to the active material structural reorganizations which occur as a consequence of the change in the lithium content in the lattice structure during Li (de)intercalation. As a detrimental side effect, they are associated with mechanical fracture of the electrode active materials, which is one of the main phenomena limiting the lifetime and long‐term performance of lithium‐ion batteries. In order to understand the dilation behavior of the NMC‐ref and LTO‐coated NMC811 electrodes, operando dilatometry was performed during galvanostatic charge and discharge. Due to the sensitivity of the dilatometry to the internal and ambient parameters such as electrode porosity, binder, electrolyte induced processes, gas evolution, electrochemical technique, rest time, and temperature, all the samples were prepared and tested under similar conditions.

**Figure 8:**
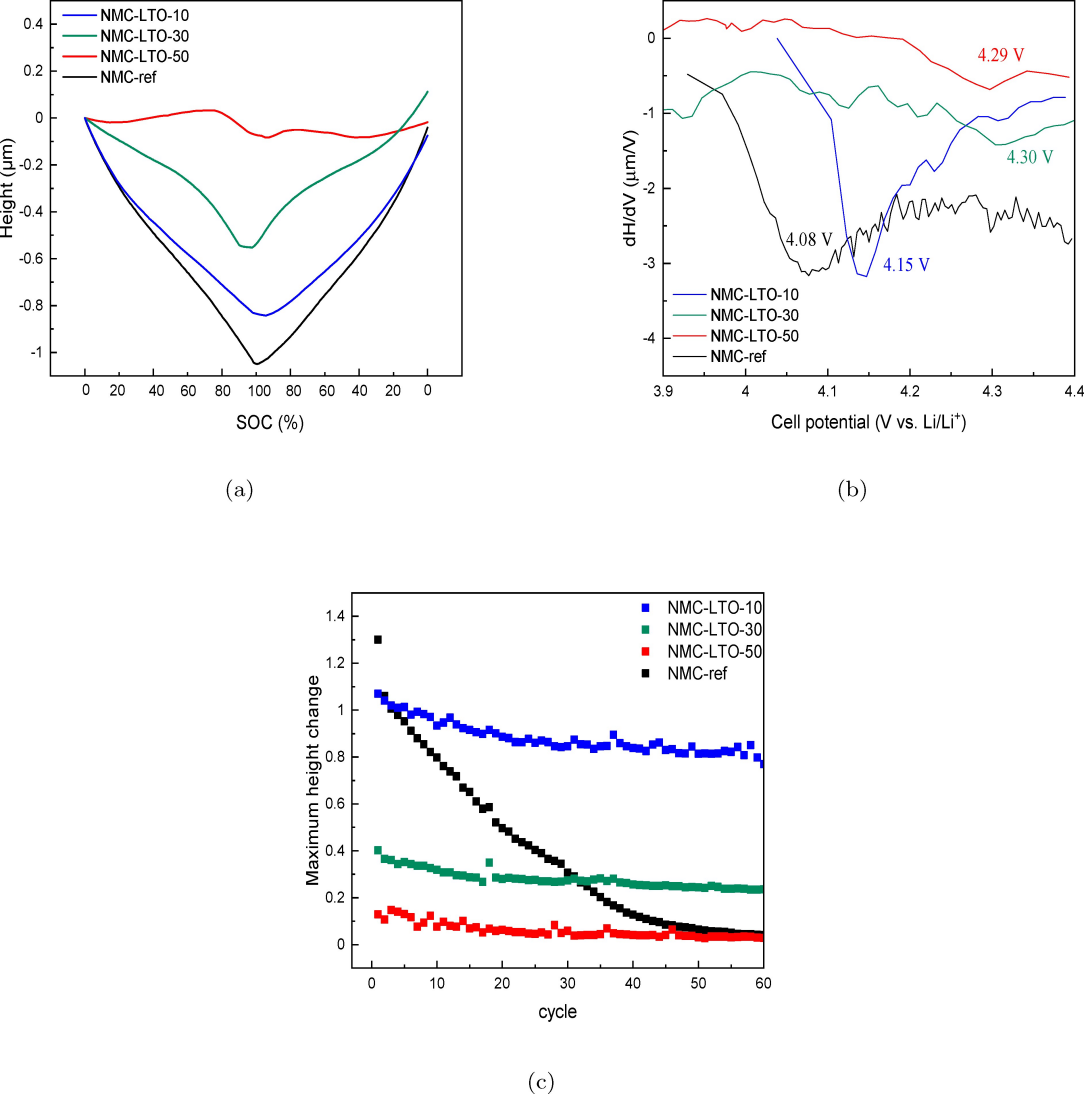
(a) Height change of the NMC, LTO‐10, LTO‐30 and LTO‐50 electrode at the initial cycles at 1 C versus experimental SOC in % in the voltage range 3.0–4.4 vs. *Li*/*Li* ^+^; (b) Differential height changes of the uncoated and LTO coated nickel‐rich positive electrodes during charging at 1C between 3.0–4.4 V charge‐discharge; (c) Maximum height change (μm) behavior of the positive electrodes during 60 cycles at 0.5 C‐rate in the voltage range of 3.0–4.4 vs. *Li*/*Li* ^+^.

**Figure 9:**
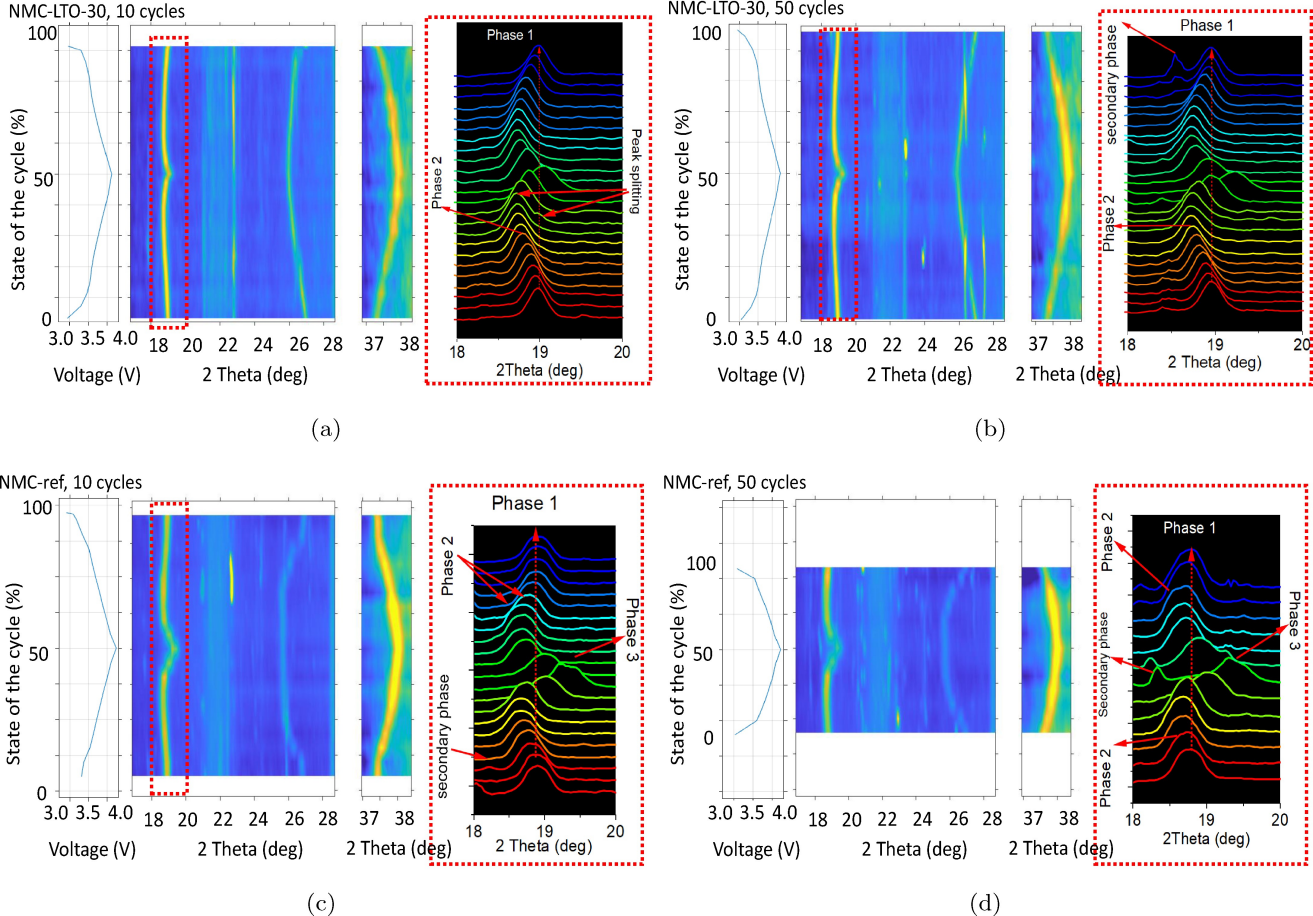
Electrochemical charge/discharge curve at 0.1 C between 2.9 and 4.3 V in a full cell, for (a–b) NMC‐LTO‐30 and (c–d) NMC‐ref after 10 and 50 cycles at 1C, aligned with the evolution of structure and phase changes during the electrochemical process for the (003) and (101) reflection.

As depicted in Figure 8a the charging process results in a noticeable change in the height change (or electrode thickness) in all the investigated samples. As lithium is extracted during charging, the thickness of the NMC811 electrodes tends to decrease. However, this change is more prominent in the NMC‐ref sample compared to the LTO‐coated NMC811 electrodes. Furthermore, all samples experience a sudden shrinkage at higher voltage ranges during charging, which is attributed to lithium extraction from the interslab sites. The magnitude of this shrinkage varies depending on the thickness of the LTO coating, with the NMC‐LTO‐50 sample exhibiting the smallest shrinkage of only 0.21 %. This highlights the importance of LTO coating thickness in controlling volume variations during charging and minimizing the risk of mechanical fracture. The initial thickness of the calendered NMC811 electrodes is 40±5 μm, and the thickness change during charging for the different samples equals to 1049 nm (2.62 %), 843 nm (2.10 %), 553 nm (1.38 %), and 83 nm (0.21 %) for the NMC‐ref, NMC‐LTO‐10, NMC‐LTO‐30, and NMC‐LTO‐50, respectively. These results indicate that the electrode thickness variation during charging reduces with the increasing LTO coating thickness. The differential analysis in Figure 8b offers an additional perspective revealing the contraction rate of the composite electrodes during the charging and the impact of the LTO coating thickness on the intensity and potential range where the contraction occurs. The highest intensity of the contraction for the NMC‐ref occurs at 4.08 V versus *Li*/*Li* ^+^, while the LTO coating induced positive shift in the contraction potential is more notable for the electrodes with the thicker LTO coating. The turning point values equal to 4.15 V, 4.30 V and 4.29 V vs. *Li*/*Li* ^+^ for NMC‐LTO‐10, NMC‐LTO‐30 and NMC‐LTO‐50, respectively. These differences in contraction at high potentials can be explained by a greatly reduced *c*‐axis evolution of the hexagonal lattice being suppressed or occurring at higher voltages when the NMC811 is modified by the LTO coating (Section 3.4, Figure 9). Spingler et al. have found that the trend of the NMC811 electrode thickness change, as measured by dilatometry, is best aligned with the *c*‐axis length variations.^[56]^ According to their analysis, the magnitude of the changes differs from one kind of an electrode to another because of absorption of active material particles expansion by the electrode structure, reduction in porosity, and non‐preferential orientation of the grains.^[56]^ As such, our results confirm that the LTO coating layer on NMC811 electrode can effectively postpone the onset of volume change and *c*‐axis contraction to higher potentials, as previously observed in other studies.[^48^, ^57^] This can contribute to higher cycling stability and highlights the potential of using coatings to control composite electrode behavior.^[19]^

Galvanostatic charge and discharge cycling using dilatometry was performed to investigate the maximum height change during each cycle for the NMC‐ref and LTO‐coated NMC811 electrodes (Figure 8c). The uncoated NMC811 sample shows drastically decreasing height changes from 1.30 to 0.02 μm during the cycling while the coated NMC‐LTO‐10, NMC‐LTO‐30 and NMC‐LTO‐50 samples are more stable. Among the coated samples, NMC‐LTO‐10 experiences the highest height change decrease from 1.07 to 0.77 μm during the 60 charge discharge cycles. NMC‐LTO‐50 and NMC‐LTO‐30 experience lower height change decreases (from 0.13 to 0.030 and from 0.40 to 0.23 μm, respectively) while the NMC‐LTO‐50 sample shows the lowest cell expansion during the cycling. The height change suppression clearly decreases with the increasing LTO coating thickness indicating that the coating limits thickness change but also hinders Li (de)intercalation during the cell cycling as discussed above. The former is reflected as improved rate capability in long‐term cycling (Figure 4) while the latter manifests itself as higher overvoltages (Figure 5) and increased impedance (Figure 6) detected for the LTO coated NMC811 electrodes. Figure 8c depicts that NMC‐LTO‐30 and NMC‐LTO‐50 demonstrate greater stability in both initial maximum height change and total height change over 60 cycles. Moreover, Figure 8c shows that although the thinnest LTO coating still improves the height change stability in comparison to NMC‐ref and yields a lower total height change in comparison to NMC‐ref, it does not provide proper protection to the surface and GBs allowing the NMC811 electrode to undergo high‐thickness changes. The thicker LTO coatings, on the contrary, evidently successfully protect the NMC811 electrode and thus their degradation during the long‐term cycling is diminished. NMC‐LTO‐30 provides superior stability as the 8–10 nm LTO coating suppresses volume changes while allowing adequate lithium transport (Figure 8). Hu et al.^[58]^ have earlier claimed that the irreversible, anisotropic, and mesoscale expansion/shrinkage of nano‐grains during the first cycle can be the dominant driving force for microcracks initiation at the GBs. Their operando XRD measurements confirm that the irreversible lattice and morphological changes induced microcracks. As the coated samples have a more rigid structure, excessive thickness changes are prevented and detrimental effects hindered as evidenced by improved mechanical integrity for the cycled LTO coated electrodes in our post‐mortem analysis (Figure 7). Thus, our results reveal that the higher stability is interlinked with suppressed mechanical deformations and diminished undesired electrode volume changes. However, the optimum thickness plays a crucial role when considering the enhancement of the electrochemical properties.

It is noteworthy that caution should be applied when making conclusions based on the dilatometry measurements as in the dilatometry setup the studied samples can excessively expand allowing higher *Li* ^+^ extraction during delithiation. In the coin cell assembly, the rigid setup limits the significant volumetric expansion of the uncoated NMC811 sample to lower values and hence the capacity and capacity retention values are different in the dilatometry and coin setup (Figures 4b and S5). The reason for lower capacity retention in dilatometry has been previously attributed to the lower applied pressure during the cell operation.^[55]^

Thus, our results reveal that the higher stability is interlinked with suppressed mechanical deformations and diminished undesired electrode volume changes. However, the optimum thickness plays a crucial role when considering the enchantment of the electrochemical properties. Hence, NMC‐LTO‐30 provides superior stability as the 8–10 nm LTO coating suppresses undesired volume changes during battery charging and discharging while allowing sufficient transport of lithium ions to maintain electrochemical properties. (Figure 8).

### Operando XRD Analysis

Based on the dilatometry analysis, the bulk phase transitions can be reasonably assumed to be restrained by the LTO coating at the surface and GBs, thus alleviating surface irreversible phase change evolution, crack formation, and resulting capacity fade. To confirm this, crystal structure changes during the Li ions (de)intercalation process were further studied using full cells with graphite negative electrodes. As depicted above (Figure 1), both the coated and uncoated NMC811 materials have a hexagonal layered *α*‐${\mathrm{NaFeO}}_{2}$ structure before cycling while the surface and GB coating neither affect the crystal structure nor can be detected. The operando XRD studies were performed on pouch cells with the NMC‐ref and NMC‐LTO‐30 positive electrodes as the latter appears to be the optimum sample. The cells were cycled at 1C, 10 and 50 times for aging, while the operando XRD was taken during a 0.1C cycle each hour. The selected 2θ regions for both samples are shown in the contour plot (Figure 9a–d) including the (003) and (101) diffraction planes. The parallel charge‐discharge curves depicted in Figure 9 for each sample show the cycling voltage range and the state of the cycle starting with charge (state of cycle=0 %) and ending with discharge (state of cycle=100 %) points. The reflection from the hexagonal (003) planes at the 2θ of 18.90° represents NMC811 *c*‐axis composed of the transition metal‐layer (${\mathrm{TMO}}_{6}$ ) and Li‐layer (${\mathrm{LiO}}_{6}$ ), whereas the (101) reflection at the 2θ of 37.30° is sensitive to the *a*‐axis.[^59^, ^60^] Figure 9a shows the XRD results for NMC‐LTO‐30 after 10 aging cycles, in which the electrode material exhibits no phase change at the very beginning of the charge. With further charging up to 4.0 V, a two‐phase co‐existence is observed (phase 2). This indicates a non‐equilibrium process caused by different Li diffusion rates in the two hexagonal phase co‐existence region.^[12]^ Following extended cycling up to 50 cycles, a comparable pattern to that of the prior NMC‐LTO‐30 is apparent. The operando XRD analysis of NMC‐LTO‐30 after 50 cycles indicates the coexistence of two phases within the same region, with no additional phase changes observed in this sample at higher SOCs. Moreover, this sample shows a symmetric structural evolution upon the initial cycles as most of the diffraction peaks return to their original positions after the lithiation.[^60^, ^61^] Meanwhile, a new peak (near 18.5°) appears at the end of the discharges which could be related to the evolution of ${\mathrm{Li}}_{2}{\mathrm{CO}}_{3}$ due to electrolyte degradation.^[62]^ For NMC‐ref (Figure 9c–d), the (003) peak shifts towards lower angles, at the initial charging state with the increase in the *c*‐parameter.[^12^, ^59^, ^60^] In addition, this peak shows clear peak broadening, suggesting significant microstrain emergence upon delithiation.^[63]^ This is further verified by the above SEM postmortem analysis and dilatometry results which suggest that stress and strain in NMC‐ref are much larger than in NMC‐LTO‐30 during the cycling. The two hexagonal phase co‐existence region after 4.0 V is not clear in NMC‐ref as the peaks overlap due to the peak broadening. However, the co‐existence can be detected as the presence of the peak splitting during the discharge as shown in Figure 9c (phase 2). These results also confirm that the phase co‐existence process is reversible, and this behavior is in accordance with the complex phase transitions, occurring during the delithiation and lithiation of ${\mathrm{LiNiO}}_{2}$ .^[41]^ With further charging the NMC‐ref cell, the (003) reflection rapidly shifts towards higher angles due to the phase transition where the lattice rapidly shrinks as a critical amount of Li is deintercalated. As shown in Figure 9c, this phase transition at high SOC includes peak splitting to another (003) signal at higher 2θ angles and distinctly shows two‐phase transitions occurring during the electrochemical (de)lithiation of NMC‐ref. This phase cannot be clearly observed in the NMC‐LTO‐30 cell, even at a high voltage of 4.2 V, suggesting that the coating suppresses the lattice contraction and the phase change is reversible during the cycling. With further cycling of the cells, the XRD diffraction patterns were measured after 50 cycles. NMC‐ref undergoes phase transformation, similarly as observed after the 10th cycle. Figure 9d shows that above 4.1 V the (003) peak of NMC‐ref markedly shifts to higher angles while splitting into two‐phase, which is ascribed to the hexagonal phase transition. Contrary to the uncoated reference electrode, this undesirable hexagonal two‐phase coexistence cannot be clearly observed for the coated NMC‐LTO‐30 even at a high voltage of 4.2 V, suggesting that the coating suppresses the change during the cycling. The evolution of the irreversible and inhomogeneous phase in NMC‐ref renders recovering the initial state after a full discharge impossible. Thus, NMC811 experiences a significant capacity fade and suffers from overvoltage increment due to structural degradation. These results are well in accordance with the dQ/dV curves illustrating the electrochemical behavior in the half cells setup (Figure 5). The irreversibility is evident for NMC‐ref as indicated by decreasing oxidation peak intensity at 4.2 V during the cycling. However, NMC‐LTO‐30 has a relatively weaker oxidation peak at 4.2 V in the beginning and this peak is not degraded by cycling, confirming the impact of coating modification toward the stabilization of the structure. The importance of the nanoscale LTO coating hence lays on its ability to effectively suppress the structural change in NMC811 while the lithium content varies and thus restraining irreversible phase changes.

**Figure 10:**
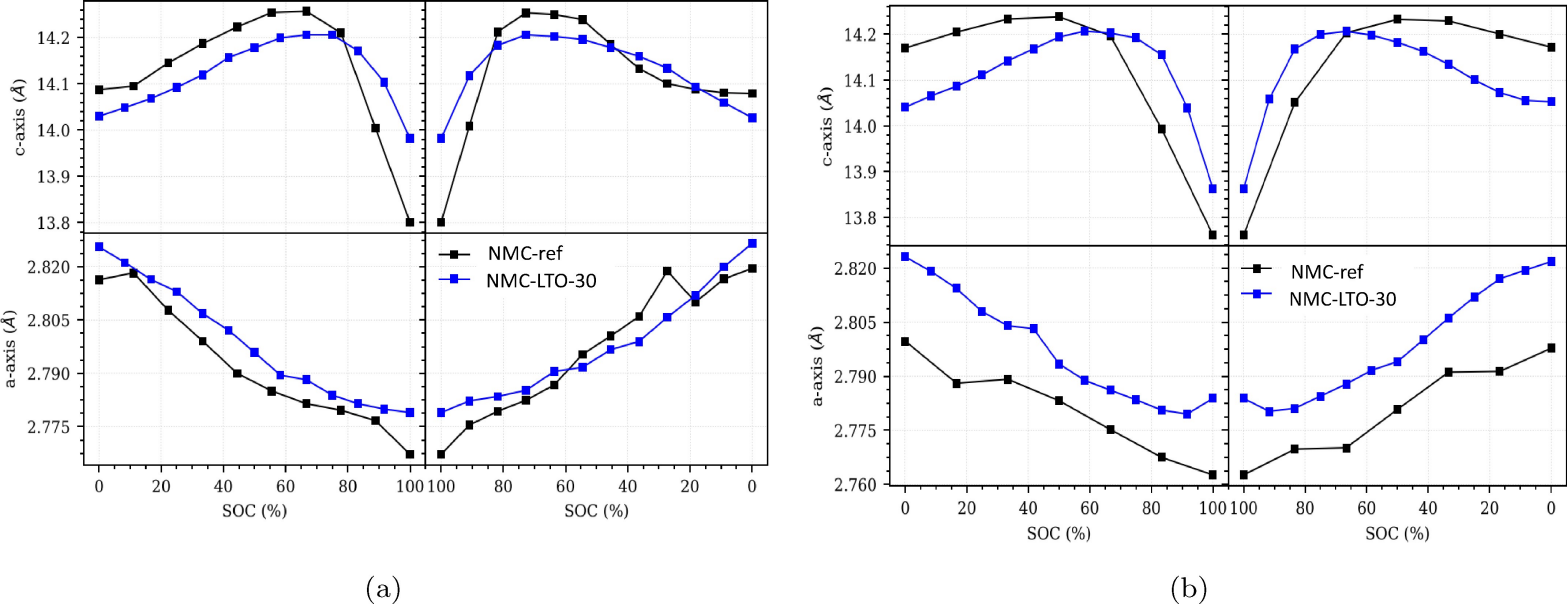
(a) Refined structural parameters for NMC‐ref and NMC‐LTO‐30 after 10 and (b) 50 cycles as a function of state of charge (%).

During the long‐term cycling, progressive structural changes in the active material adversely accumulate resulting in structural degradation and further capacity fading.[^12^, ^60^, ^64^, ^65^] Figure 10a–b summarizes the changes of the *c*‐axis and *a*‐axis lattice constants as a function of the SOC upon a full charge discharge cycle of NMC‐ref and NMC‐LTO‐30. Generally, during the charging process, the lattice parameter *c* gradually increases when the Li ions deintercalate from the Li layers, thus increasing the electrostatic repulsive force in the neighboring oxygen slabs.^[59]^ With further charging of the cell, the *c*‐axis first continuously increases for both NMC‐ref and NMC‐LTO‐30 whereas its dramatic decrease is observed after SOC 70–80 %. At the end of the charging, the c‐axis of NMC‐ref drops from 14.08 to 13.80 Å while for NMC‐LTO‐30 the decrease is notably smaller, from 14.03 to 13.98 Å. For the former, the dramatic change of the c‐axis at the higher potentials leads to irreversible phase change due to oxygen release from the NMC structure.^[66]^ As lithium ions are removed from the positive electrode in lithium‐ion batteries, the transition metal ions in the electrode become more oxidized. This leads to an increased attraction between the transition metal ions and the oxygen atoms in the TM−O bond. As a result, the electron density on the oxygen atoms decreases, leading to a distortion in the lattice structure of the electrode. This distortion can reduce the electrostatic repulsion between ${\mathrm{TMO}}_{6}$ slabs and cause a decrease in TM slab thickness. This effect is common in transition metal oxides and is important to understand and control to optimize the performance and stability of these materials in battery applications. Thus, if the lattice distortion becomes too severe, it can lead to mechanical stress and structural damage to the active material, potentially triggering the release of oxygen.[^56^, ^58^, ^67^] This also can cause the cracking and poor connections between particles in the electrode after extensive cycling, which might be one of the reasons for the poor cycling performance of the NMC‐ref cells after long‐term cycling between 3.0–4.4 V. As such, the GB and surface coating enhances the structural stability, decreases the volume change, and suppresses phase transitions, resulting in less surface oxygen escape, crack formation, and capacity fade.

## Conclusions

In summary, the development of high‐performance and stable electrode materials is crucial for the next‐generation LIBs, which are used in a wide range of applications. In this study, a surface and GB coating with LTO was successfully applied onto NMC811 electrodes through an ALD process. The systematic investigation of the LTO‐coated NMC811 and NMC‐ref structural stability and electrochemical performance was done by STEM, SEM, operando XRD, galvanostatic charge‐discharge, and dilatometry techniques which demonstrate the effectiveness of the LTO coating in enhancing the stability and performance of the Ni‐rich electrode material. The obtained results reveal that the LTO coating effectively stabilized the NMC811 active material by hindering the electrode height change which is found to be 2.62 % for NMC‐ref and 2.10 %, 1.38 %, and 0.21 % for NMC‐LTO‐10, NMC‐LTO‐30, and NMC‐LTO‐50, respectively. This result highlights that the chemomechanical degradation, intergranular crack formation, and anisotropic volume changes, are suppressed in LTO coated sample leading to prolonged stability and maintained performance. The reversible capacity of the LTO‐coated electrodes after 100 cycles correspond to a high retention of above 93 %, while for the uncoated NMC811 it decreased to 86 %. During the long‐term cycling, progressive structural changes in the active material adversely accumulate resulting in structural degradation and further capacity fading. However, the highly stable LTO‐coated NMC811 electrode shows a symmetric structural evolution upon the cycling and most of the diffraction peaks return to their original positions after the lithiation. The study highlights the importance of the GB coating in enhancing the stability of the Ni‐rich electrode material and demonstrates the LTO coating significance as a protective coating on the electrode surface and GBs. Therefore, this research provides valuable insights into the development of high‐performance and stable electrode materials for future LIBs, contributing to the progress toward the development of more efficient and sustainable energy storage systems.

## Experimental

### Electrode Preparation

NMC811 precursors for synthesizing positive electrode active material were provided by Umicore Finland Oy. In order to synthesize the cathode active material, 0.06 mol of the NMC precursor was mixed uniformly with 0.06 mol of LiOH (Thermo Fisher, 98 %). The mixture was sintered at 800 °C for 12 hrs in a tube furnace (R70/9, Nabertherm) in an oxygen atmosphere while heating rates were 3 °C/min. After cooling down to room temperature, the cathode powder was ground to ensure that the material did not contain any agglomerates. To fabricate electrodes, a slurry was first prepared and then uniformly laminated on an aluminum foil resulting in the total loading between 5.2–7.0 mg/cm^2^ after drying. The NMC811 electrode laminates contained 93 wt % active material (NMC811), 3 wt% carbon black (Timcal Super C65), and 4 wt % polyvinylidene fluoride (PVDF, Solvay, Solef 5130). Graphite electrodes were prepared by coating a slurry on the surface of a Cu foil. The negative electrode consisted of 92 wt % graphite (Imerys graphite and carbon, C‐NERGY), 2 wt % carbon black, and 6 wt % PVDF with the total loading between 6.5–8.4 mg/cm^2^ after drying. All electrodes were dried in an oven at 80 °C for 4 h prior to cutting into 14, 18, and 10 mm diameter discs for coin cell, EL‐Cell, and dilatometry studies respectively, and were calendered with a pressure of 3250 kg/cm^2^ at room temperature. The prepared electrodes were dried in a vacuum oven for 12 h at 80 °C and subsequently transferred to an argon‐filled glovebox (Jacomex, with oxygen and water levels below 1 ppm) before assembling.

### Electrode Coating

The ${\mathrm{Li}}_{x}{\mathrm{Ti}}_{y}O_{z}$ ALD (LTO) process for coating the Ni rich electrodes, has been developed in our previous studies.^[19]^

In this work, the LTO coating was deposited on the prefabricated NMC811 laminates with a commercial flow‐type hot‐wall ASM F‐120 ALD reactor at 200 °C and below 3 mbar pressure. The LTO‐ALD films were deposited using titanium tetra‐isopropoxide (TTIP, Aldrich 97 %Ti[OCH(CH)_2_]_4_), lithium *tert*‐butoxide (LTB, Aldrich 97 % (CH_3_)3COLi), and water as precursors. To produce a sufficient vapor, TTIP and LTB were evaporated at 40 °C and 170 °C, respectively, from open glass boats inside the reactor. Nitrogen (99.999 %) was used as a precursor carrier and purge gas. The LTO layer was deposited by using 10, 30 and 50 supercycles while each supercycle consisted of four binary cycles of ${\mathrm{TiO}}_{x}$ and one binary cycle of ${\mathrm{Li}}_{x}O$ /LiOH (Li/Ti=$\frac{1}{4}$ ). The TTIP precursor and LTB were separated by a long $N_{2}$ purging period. The pulse/purge times for TTIP and LTB were 9/10 s and 3/10 s, respectively. Water pulse/purge for both TTIP and LTB was 0.5/15 s. The growth per each binary cycle (GPC) was 0.4 Å, and the GPC of the LTO supercycle was 2 Å. Since the GPC remains constant at a set temperature during ALD‐type growth, we increased the number of cycles to increase thickness for the LTO‐coated NMC811 electrode. To differentiate these LTO‐coated NMC electrodes, we named them based on the number of ALD cycles NMC‐LTO‐10, NMC‐LTO‐30, and NMC‐LTO‐50 referring to 10, 30 and 50 supercycles, respectively. Accordingly, the bare (uncoated) NMC811 electrode is denoted as NMC‐ref. Additionally, the chemical composition of the ${\mathrm{Li}}_{x}{\mathrm{Ti}}_{y}O_{z}$ coating on the silicon wafer was analyzed using time‐of‐flight elastic recoil detection analysis (TOF‐ERDA). This technique allows for the quantitative depth profiling of all atoms in the sample, including those with low atomic numbers or electron density such as lithium. Based on the results, the deposited film contains both lithium and titanium. The ratio of Li/Ti remains constant throughout the film thickness and is approximately 0.9, confirming a chemical composition similar to ${\mathrm{Li}}_{4}{\mathrm{Ti}}_{5}O_{12}$ .^[19]^

### Structural Characterization

Coated and uncoated NMC electrodes were analyzed using scanning electron microscope (SEM, Tescan Mira3‐SEM JEOL JIB‐4700F) coupled with an energy‐dispersive X‐ray spectroscopy (EDS) detector for their morphological characteristics and determining element distribution. The operation voltage was 10 kV for SEM and 20 kV for EDS analysis. The crystal structure of the coated electrodes was characterized by ex‐situ X‐ray diffraction (XRD) analysis with a PANalytical X′Pert Pro MPD Alpha‐1 diffractometer using Cu K$\alpha_{1,2}$ ‐radiation with a wavelength of 0.154 nm (operation voltage 45 kV, current 40 mA). The measurements were done in a 2*θ* range of 5–80° with steps of 0.026°. The structural and elemental evolution of NMC811 electrodes were studied using focused‐ion beam (FIB) cutting and transmission electron microscopy (TEM). FIB processing was carried out by using a JEOL JIB‐4700F instrument in which a high‐current density Ga ion beam was employed for fast ion milling and processing of specimens. The FIB was used to cut the cross‐section of the coated NMC electrodes and prepare the lamella for TEM analysis. Prior to FIB cutting, a Pt layer with 1 μm thickness was deposited on the surface of the samples to protect against ion beam damage during the preparation. The cross‐sectional structure of the prepared samples was investigated by a JEOL JEM‐2200FS double aberration‐corrected microscope equipped with a 200 kV field‐emission gun (FEG) and X‐ray EDS detector. Operando X‐ray diffraction (XRD) was conducted for pouch cells prepared for uncoated and coated NMCs. The specifics of the sample preparation are described in *Electrochemical investigation* section. The XRD patterns were measured using a wide‐angle x‐ray scattering (WAXS) set up. The radiation from a water‐cooled Cu anode x‐ray tube (36 kV and 40 mA) was monochromated and the Cu K$\alpha_{1,2}$ ‐radiation was reduced to a size of 0.5 mm×0.5 mm using slits. The x‐rays scattered by the cell were detected in transmission mode using a MAR345 image plate with 0.15 mm pixel size and 345 mm diameter. The distance between the sample and the detector was 143 mm, and the scattering angle range achieved with this setup was 1–52° limited by the beam stop (low limit) and detector size (high limit). The resolution as determined from the full width at half maximum of the XRD peaks was about 0.1° and the step size of the radial integration was 0.03°. In an XRD measurement, the cell was charged or discharged at 0.1C, and the exposure time for one WAXS image was 1 hour. The XRD patterns were integrated from the WAXS images using a radial integration as implemented in the software FIT2D.

### Electrochemical Investigations

Coin cells CR‐2016 (Hohsen) were assembled in an Ar‐filled glove box after LTO coating. We conducted electrochemical investigations for the coated and uncoated samples to compare their cycling performance. Cell assembly was carried out with lithium‐metal foil discs with 19 mm diameter (0.74 mm thickness, Alfa Aesar) as the anode and fiber glass discs (GF/A 0.26 mm, Whatman) as the separator. The electrolyte was 1 M ${\mathrm{LiPF}}_{6}$ in ethylene carbonate (EC)/ethylmethyl carbonate (EMC) (1 : 1 by weight, BASF, LP30). After cell assembly, all the cells were allowed to rest for 24 h prior to their electrochemical studies at room temperature. Galvanostatic charge‐discharge was carried out using a Land battery test system and the cells were subjected to rate capability measurements with various C‐rates (1 C=200 mA/g). In addition, samples were cycled at 1C for long‐term cyclability via a constant current (CC−CV) mode in the voltage range of 3.0–4.4 V. At least two parallel samples were measured to confirm the repeatability of the results. Electrochemical impedance spectroscopy (EIS) measurements were carried out in the frequency range of 100 kHz to 10 mHz with an amplitude voltage of 5 mV (Biologic VSP‐3e P/Gstat potentiostat) using an EL‐CELL ECC‐Combi three‐electrode cell setup (with Li metal as a counter and reference electrode). All EIS measurements were done at an open‐circuit voltage corresponding to the 50 % state of charge (SOC), and Zview software was used for spectral fitting. Dilatometry analysis was done with an ECD‐3‐nano (EL‐CELL) dilatometer to investigate the operando volume change of the NMC and LTO‐coated NMC electrodes with different coating thicknesses (10 mm diameter). In this setup, lithium foil was used as a counter electrode, and a glass frit separator prevented the height change effect of the counter electrode. The positive electrodes were cycled between 3.0 and 4.4 V versus *Li*/*Li* ^+^. The dilatometry measurements were performed inside a climatic chamber (VC3 4018) with a fixed temperature of 25 °C.

Pouch cell assembly of the coated and uncoated NMC samples was used to perform the operando XRD studies to compare their structural behavior. Pouch cell assembly was carried out with graphite as a negative electrode and ceramic‐coated polymer film (Gelon) as a separator. The size of the graphite electrode was 28 mm×18 mm, and the NMC and coated NMC electrodes were 27 mm×17 mm. The separator size was 30 mm×18 mm and 1 M ${\mathrm{LiPF}}_{6}$ in 25 : 70 : 5 ethylene carbonate (EC)/diethylene carbonate/propylene carbonate solution with 1 mol% vinylene carbonate was used as an electrolyte (E‐lyte). After the cell assembly, the pouch cells were allowed to rest for 24 h and subsequently formatted by cycling at 0.1C (three times) prior to galvanostatic charge‐discharge at room temperature.

## Conflict of Interests

The authors declare no competing financial interest.

1

## Supporting information

As a service to our authors and readers, this journal provides supporting information supplied by the authors. Such materials are peer reviewed and may be re‐organized for online delivery, but are not copy‐edited or typeset. Technical support issues arising from supporting information (other than missing files) should be addressed to the authors.

Supporting Information

## Data Availability

The data that support the findings of this study are available from the corresponding author upon reasonable request.
