# Supplementary material for: Surface and Grain Boundary Coating for Stabilizing LiNi0.8Mn0.1Co0.1O2 Based Electrodes
Source: ChemSusChem. 2024 Aug 12;17(23):e202400272. doi: 10.1002/cssc.202400272 (PMC11632585; doi:10.1002/cssc.202400272)
Supplement: Supplementary file 1 — Supporting Information [file CSSC-17-e202400272-s001.pdf]

ChemSusChem

Supporting Information

Surface and Grain Boundary Coating for Stabilizing $LiNi_{0.8}Mn_{0.1}Co_{0.1}O_2$ Based Electrodes

Zahra Ahaliabadeh, Ville Miikkulainen, Miia Mäntymäki, Seyedabolfazl Mousavihashemi, Lide Yao, Hua Jiang, Simo Huotari, Timo Kankaanpää, Tanja Kallio,* and Mattia Colalongo

Surface and grain boundary coating for stabilizing $LiNi_{0.8}Mn_{0.1}Co_{0.1}O_2$ based electrodes

Zahra Ahaliabadeh,[†] Ville Miikkulainen,[†] Miia Mäntymäki,[‡] Seyedabolfazl Mousavihashemi,[¶] Yao Lide,[§] Hua Jiang,[§] Simo Huotari,[‖] Timo Kankaanpää,[⊥] and Tanja Kallio[*,†]

†*Department of Chemistry and Materials Science (CMAT), School of Chemical Engineering, Aalto University, 02150, Espoo, Finland*

‡*Department of Chemistry, University of Helsinki, 00014, Helsinki, Finland*

¶*Department of Chemistry and Materials Science, School of Chemical Engineering, Aalto University, 02150, Espoo, Finland*

§*Department of Applied Physics, School of Science, Aalto University, 02150, Espoo, Finland*

‖*Department of Physics, University of Helsinki, 00014, Helsinki, Finland*

⊥*Umicore Finland Oy, 67101 Kokkola, Finland*

E-mail: tanja.kallio@aalto.fi

Phone: +358 50 5637 567

1 Characterization analysis

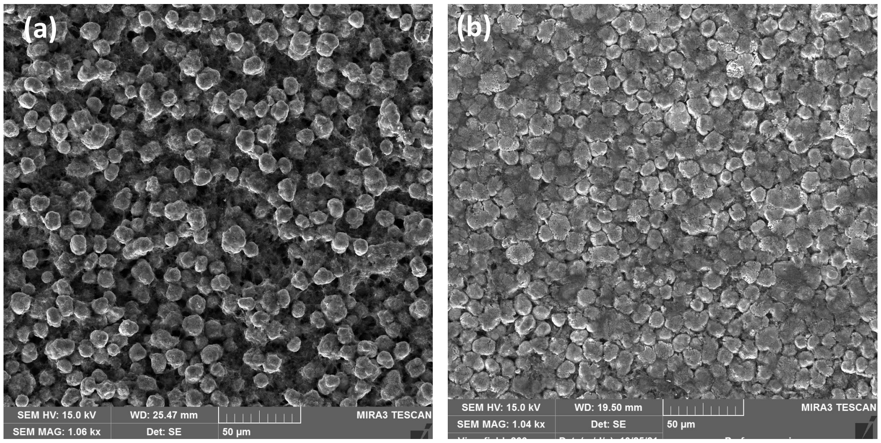

Figure 1S: Difference between (a) the non-calendered and (b) calendered NMC811 electrode morphology.

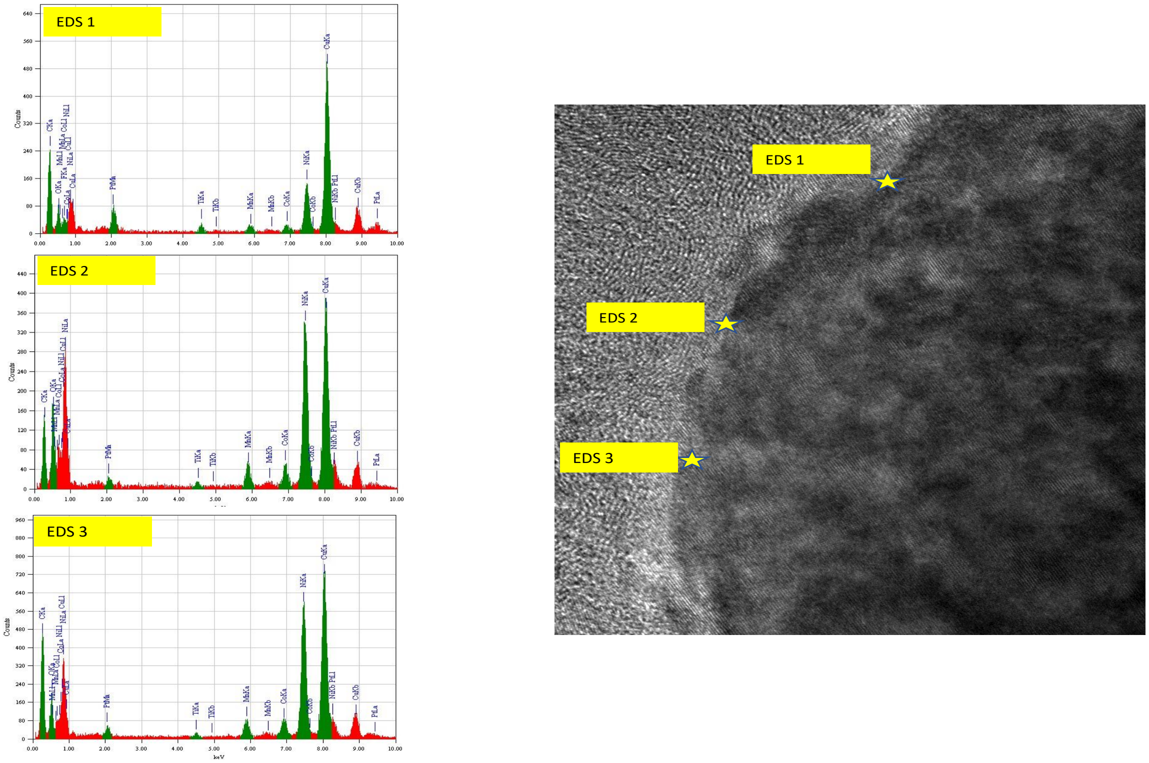

Figure 2S: A TEM image and representative EDS analysis for the NMC-LTO-10 samples

2 Electrochemical analysis

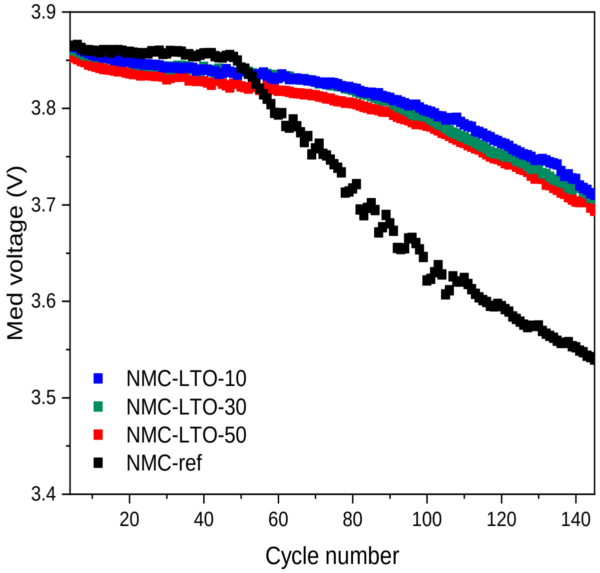

Figure 3S: Discharge voltage decay of the NMC-ref, NMC-LTO-10,NMC-LTO-30 and NMC-LTO-50 electrodes at 1 C in half cell when cycled at the voltage range of 3.0–4.4 V.

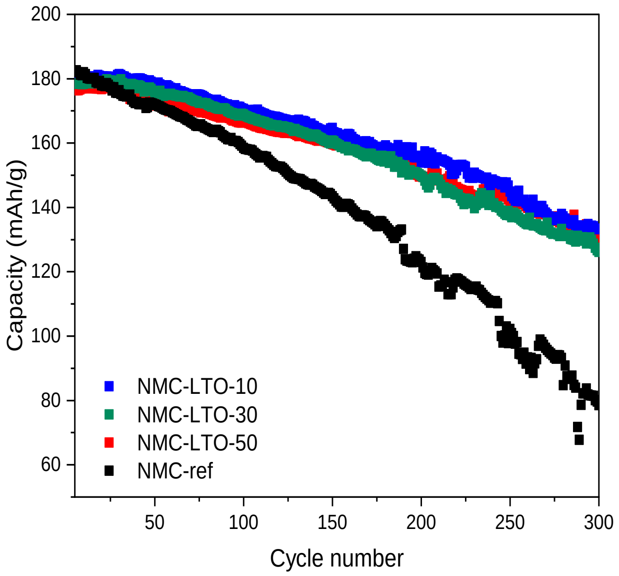

Figure 4S: Long-term cyclability (charged and discharged at 1 C) of the NMC-ref, NMC-LTO-10, NMC-LTO-30 and NMC-LTO-50 electrodes in the voltage range of 3.0–4.4 V for 300 cycles in half cells.

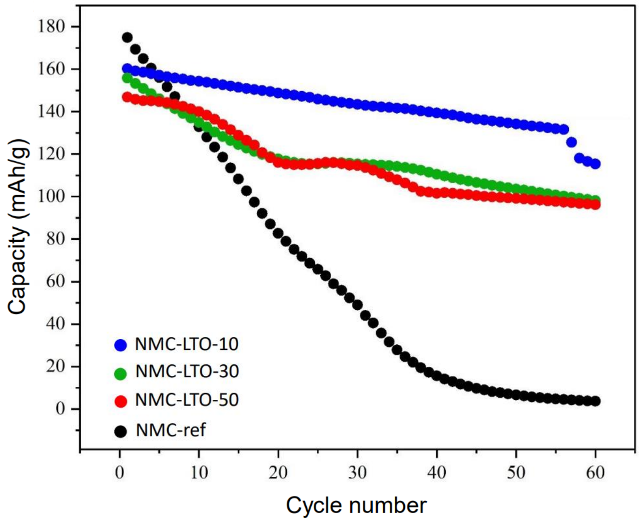

Figure 5S: Discharge capacity of the NMC-ref, NMC-LTO-10, NMC-LTO-30 and NMC-LTO-50 electrodes during cycling at 0.5 C in the 3.0-4.4 V vs. Li^{+}/Li potential window.
